# Anti-inflammatory and Anti-infectious Dietary Paradigms May Be Crucial for Visceral Weight Reduction

**DOI:** 10.3389/fimmu.2019.00422

**Published:** 2019-03-08

**Authors:** Dong-Mei Chen, Meng-Le Zhang, Zhu-Qing Shi, Chang-Qing Li, Qi Wang, Jian-Ping Song, Qin Xu, He Li, Qing-Ping Zeng

**Affiliations:** ^1^Institute of Tropical Medicine, Guangzhou University of Chinese Medicine, Guangzhou, China; ^2^School of Basic Course, Guangdong Pharmaceutical University, Guangzhou, China; ^3^Science and Technology Industrial Park, Guangzhou University of Chinese Medicine, Guangzhou, China; ^4^Institute of Clinical Pharmacology, Guangzhou University of Chinese Medicine, Guangzhou, China

**Keywords:** obesity, weight loss, inflammation, infection, nutritional immunology

Based on body mass index (BMI), body weight has been classified into overweight (25–29.9 kg/m^2^), obesity (≥30 kg/m^2^), and severe obesity (≥40 kg/m^2^) (1). BMI >30 has been classified as a disease state by the American Medical Association (AMA) (2). According to the World Health Organization (WHO), the worldwide prevalence of obesity almost tripled between 1975 and 2016. Overall, 1.9 billion (39%) and 650 million (13%) of adults aged 18 years and over were overweight and obese, respectively, in 2016 (3). From 2007–2016 in the US, middle-aged obese adults (40–59 years old) were more prevalent than obese young adults (4). These statistical data identified middle-aged obese adults as a high-risk population vulnerable to obesity-related metabolic syndrome.

Visceral obesity, also known as abdominal, central, or ectopic obesity, was defined as a waist circumference ≥102 cm in men and ≥88 cm in women (5), or as a waist-to-hip ratio >0.9 for men and >0.85 for women (6). Recently, 50% of men and 70% of women among US adults aged from 50 to 79 years were diagnosed with visceral obesity (7), which closely correlated with insulin resistance, type 2 diabetes, and cardiovascular disease (8).

Neither BMI-defined obesity nor visceral obesity provides guidance on how to reduce weight in young or elderly populations because the etiology of obesity in these populations is uncertain. We suggest that weight loss would be more practical if obesity was simply classified into inflammatory and non-inflammatory subtypes, regardless of race and gender. We believe that a shift from non-inflammatory obesity to inflammatory obesity may be aging-dependent and adipose depot-specific. As such, subcutaneous obesity in young individuals, with the exception of extreme cases of adolescent obesity, would be generally non-inflammatory. In contrast, visceral obesity frequently seen in elderly individuals is more likely to be inflammatory. However, inflammatory obesity can also occur in young adults, adolescents, and children (see below).

This classification of obesity subtypes was suggested because we believe that immunosurveillance determines conversion from non-inflammatory to inflammatory obesity subtype. Aging-driven immunosenescence deteriorates innate and adaptive immunity, leading to compromised elimination of pathogenic and opportunistic infections (9). Non-inflammatory obesity equates to metabolically-healthy obesity, whereas inflammatory obesity equates to metabolically-unhealthy obesity (10), with the latter associated with increased risk of cardiovascular disease-related mortality (11).

In general, visceral obesity is accompanied by infiltration of activated macrophages and other immunocompetent cells, as demonstrated by increased area, density, and presentation of inflammatory markers in abdominal intramuscular adipose tissue (12). Adipose inflammation is likely induced by either the bacterial endotoxin lipopolysaccharide (LPS) (13), fatty acids (14), or ceramides (15).

Visceral obesity could be conveniently measured using a bioelectrical impedance analysis (BIA)-based electric meter (16). Accordingly, lower basal metabolic rate (BMR) and lower body water rate (BWR), which can be measured using these meters, might be indicators of visceral obesity, although this association requires further elucidation. However, inflammation causes mitochondrial dysfunction, resulting in disruption of fatty acid oxidation, and decreased ATP and H_2_O production. For example, age-dependent obesity was associated with decreased mitochondrial complex IV activity, resulting in reduced fatty acid oxidation and subsequent adipocyte hypertrophy (17).

Therefore, inflammatory obesity in immunocompromised middle-aged and elderly adults is assumed to originate from gut dysbiosis, colon damage, LPS leakage, and mitochondrial depletion. This subtype of obesity, characterized by less fatty acid degradation, may be ameliorated by anti-infection and anti-inflammatory treatment.

In contrast, non-inflammatory obesity in adolescents or children with competent immune systems may simply result from excessive food intake and inadequate energy expenditure. This subtype of obesity is characterized by increased fatty acid and fat synthesis, and may be best treated by calorie restriction (CR), intermittent fasting (IF), exercise training, or other weight-reducing procedures.

## Gut Dysbiosis Induces Colon Damage and Endotoxin Leakage

Recent studies suggested that sensitivity of gut microbiota to host genetic and dietary influences contribute to risk of development of obesity and related metabolic disorders (18). A previous study showed that 37.6% of obese children presented with small intestine bacterial overgrowth (SIBO). Non-alcoholic fatty liver disease (NAFLD), hypertension, and metabolic syndrome accounted for 59.5, 23.4, and 44.6% in the SIBO positive group, compared with 10.2, 5.1, and 9% in the SIBO negative group (19), implying that intestinal infection was a major contributor to NAFLD, hypertension, and metabolic syndrome in obese children.

A common ingredient in livestock and poultry products, chondroitin sulfate (CS), increases abundance of *Bacteroides thetaiotaomicron*, a species of sulfatase-secreting bacteria that degrades mucins to supply sulfate to *Desulfovibrio piger*, a species of sulfate-reducing bacteria (20). Heme, a rich component in red meat, contributes to increased abundance of *Akkermansia muciniphila*, a species of mucus-degrading bacteria, and further facilitates aberrant colon epithelial proliferation through consumption of mucins (21). Beneficial or harmful effects of *A. muciniphila* have been shown to be abundance-dependent. Colon integrity and barrier function were reinforced by adequate mucin-consumer residence, but compromised by excessive mucin consumption (22).

High-fat diet (HFD) led to increased secretion of bile acids (BAs), followed by alterations in microbial compositions. Feeding mice BAs with a normal diet induced an obese phenotype, similar to that seen in HFD-fed mice. Interruption of BA biosynthesis attenuated HFD-shaped plasticity of the gut microbiome (23). HFD increased oxidative stress and disrupted intestinal gap junction proteins, increased membrane permeability, and contributed to endotoxemia, inflammation, and intestinal tumorigenesis (24).

## Gut Dysbiosis Triggers Adipose Inflammation and Mitochondrial Dysfunction

Mice fed an obesogenic but non-inflammatory diet developed metabolically-healthy obesity, but fed a Paigen diet developed metabolically-unhealthy obesity. This study showed that T lymphocyte infiltration occurred in response to obesogenic and Paigen diets, but CD4^+^ and CD8^+^ cells were increased only in Paigen-fed mice, and showed increased expression of interleukin 1 (IL-1), IL-4, IL-6, IL-17, and interferon γ (IFN-γ). Accordingly, the colon-destroying bacteria *Bacteroidia, Deltaproteobacteria*, and *Verrucomicrobia* dominated the gut lumen of mice fed a Paigen diet (25). These results provided direct evidence supporting classification of obesity into inflammatory and non-inflammatory subtypes.

As brown adipose tissue (BAT), which contains a large amount of mitochondria, converts to white adipose tissue (WAT), which contains relatively fewer mitochondria, many degenerating mitochondria containing activated inflammasome NLR family pyrin domain containing 3 (NLRP3) were observed in whitened adipocytes (26). Upon activation of hypoxia-inducible factor 1α (HIF-1α), palmitate-induced pro-inflammatory cytokine IL-1β and macrophage Janus kinase-p38 mitogen-activated protein kinase (JNK-p38 MAPK) were upregulated and activated (27). Activity and assembly of mitochondrial complex IV were repressed in adipocytes of middle-aged mice and human visceral adipose tissue in a HIF-1α-dependent manner (28).

When mitochondrial density becomes scattered and dysfunctional as an outcome of inflammation, fatty acids from fat digestion cannot be appropriately converted to adenosine triphosphate (ATP), CO_2_, and H_2_O. Because of mitochondrial dysfunction, inflammatory obesity should be characterized by incomplete fatty acid oxidation. Indeed, knockout of the anti-inflammatory cytokine IL10 resulted in an inflammatory state, which lowers body temperature in newborns due to impaired UCP1-dependent mitochondrial respiration in BAT (29). As further evidence, anti-inflammatory effects induced by antibiotics or non-steroidal anti-inflammatory agents (NSAIDs), such as aspirin, showed better weight-reducing effects (30).

In turn, water deficits increase serum levels of antidiuretic hormone (ADH), vasopressin, and glucocorticoids, resulting in activation of serum- and glucocorticoid-inducible kinase 1 (SGK1), adipose deposition, and obesity-related disorders. Accordingly, water insufficiency also augmented nuclear factor of activated T-cells 5 (NFAT5) effects that could stimulate SGK1 activation and induce fat deposition (31).

## An Anti-inflammatory High-Fat and Low-Carbohydrate Diet Might be Effective for Adipose Weight Loss

Anti-obesity effects exerted by a high-fat and low-carbohydrate ketogenic diet (KD) have been extensively debated (32–34) because the ketogenic diet has been shown to contribute to gut dysbiosis. Recent clinical trial data have indicated that KD represents a healthy diet for weight loss (35). A meta-analysis of 13 randomized controlled trials over 1 year indicated that volunteers on a very low carbohydrate KD tended to lose more weight than those on a low-fat diet in five trials (36). In an 8-week randomized trial that included 34 obese men and women aged 60 to 75, those on the KD lost 9.7% of body fat, while those on a low-fat diet lost only 2.1% of body fat, and those on the KD lost three times more visceral adipose weight than those on a low-fat diet (37).

In a 3-month prospective observational study of glucose transporter 1 (GLUT1) deficiency syndrome, a disorder in which individuals cannot utilize glucose, KD significantly increased *Desulfovibrio* spp., a bacterial group linked to gut mucosa inflammation and animal fat consumption (38). *Akkermansia* and *Parabacteroides* enriched by KD provided protection from seizure in a mouse seizure model (39). Interestingly, KD reversed overgrowth of *A. muciniphila* and elicited an anti-microbial-like effect in mice (40). β-hydroxybutyrate, a major ketone body (KBs) derived from fatty acid degradation in the liver, could block NLRP3-mediated inflammation and attenuate IL-1β secretion (41), implying that KD might modulate host inflammatory responses through high fat content leading to inflammation and β-hydroxybutyrate production leading to anti-inflammatory effects.

The low carbohydrate effects of KD could mimic CR to activate adenosine monophosphate-activated protein kinase (AMPK) after an increase in AMP, leading to activation of silent mating type information regulation 2 homolog-1 (SIRT1) after an increase in nicotinamide adenine dinucleotide (NAD^+^). AMPK and SIRT1 cooperatively activate peroxisome proliferator activated receptor γ coactivator 1α (PGC-1α) to induce mitochondrial biogenesis, fatty acid oxidation, and adipose weight loss (42, 43). Crosstalk between KD-mediated histone deacetylase (HDAC) inhibition and mechanistic target of rapamycin catalysis subunit 1 (mTORC1) signaling has been shown to contribute to lifespan extension in mice (44, 45).

Peroxisome proliferator activated receptor α (PPARα), a key transcription factor in regulation of ketogenesis, has been shown to participate in signaling driven by AMPK, PGC-1α, and mTORC1. PPARα also induced the hormonal mediator fibroblast growth factor 21 (FGF21) to activate hepatic lipolysis and ketogenesis (46). Therefore, a KD-like diet can prompt fatty acid conversion to anti-inflammatory KBs in the liver when glucose supply is insufficient.

## An Anti-infectious Fiber-Rich Diet Might Contribute to Adipose Weight Loss

Gut microbial fermentation of vegetables and fruits produces short-chain fatty acids (SCFAs) including acetate, propionate, and butyrate. SCFAs contributed to a healthier gut microbial ecological system and ameliorated type 2 diabetes (47). Furthermore, butyrate protected mice against methionine–choline-deficient diet-induced non-alcoholic steatohepatitis (NASH) by improving gut barrier function, attenuating inflammation, and reducing endotoxin levels (48). Butyrate also activated G protein-coupled receptor 43 (GPR43) and suppressed insulin signaling in adipocytes, thereby inhibiting fat accumulation and promoting lipid metabolism (49). Hepatic mitochondria served as the main targets of butyrate in reversing insulin resistance and blocking fat accumulation in diet-induced obese mice (50). Therefore, fiber-rich diets have anti-infectious properties, and components of these diets can be fermented into anti-infectious SCFAs by gut bacteria.

Due to being structurally related, butyrate and β-hydroxybutyrate should be functionally redundant. A recent study demonstrated that several four-carbon organic molecules, including butyrate and β-hydroxybutyrate, favored energy expenditure and alleviated oxidative stress (51). Additionally, acetate showed anti-inflammatory and oxidative stress-modulating properties in different immune cells (52), suggesting that a diet that contains vinegar may help to prohibit bacterial overgrowth, maintain gut microbiota homeostasis, and contribute to adipose weight reduction.

## An Innovative Adipose Weight-Reducing Diet for Middle-Aged Obese Adults

We strongly recommend a convenient and practical “farmer-hunter” diet, or a modified fiber-rich KD, as an ideal weight-reducing dietary option for middle-aged obese adults. This diet includes a high-fiber vegetarian breakfast and lunch (07:00–19:00 for 12 h duration) supplemented with tea and coffee, and a KD-like carnivorous dinner (19:00–07:00 for 12 h duration) combined with wine or vinegar. First, carnivorous food (meat, fish, and sea food)-derived KBs prevent chronic inflammation and mimic CR to enhance mitochondrial biogenesis for effective fatty acid oxidation and energy expenditure (53). Second, vegetarian food (cereals, legumes, vegetables, and fruits)-derived SCFAs mitigate meat-induced gut opportunistic infection and maintain gut ecological homeostasis (54). Third, tea, coffee, and wine, rich in polyphenols, can serve as anti-oxidants or can be fermented to SCFAs to prevent infection (55). Finally, acetate in vinegar can mimic SCFAs to exert anti-infectious effects (56).

Our recommendation is that rice, bread, and other starchy foods must be consumed separately from meat, fish, and seafood to avoid conversion of excess glucose to lipids. Without worrying about meat and oil-induced gut dysbiosis (21–24), those above described anti-inflammatory and anti-infectious dietary components should ensure sustainable availability of active and functional mitochondria for fatty acid metabolism and adipose weight loss (31–36).

## Conclusion

**Figure 1:**
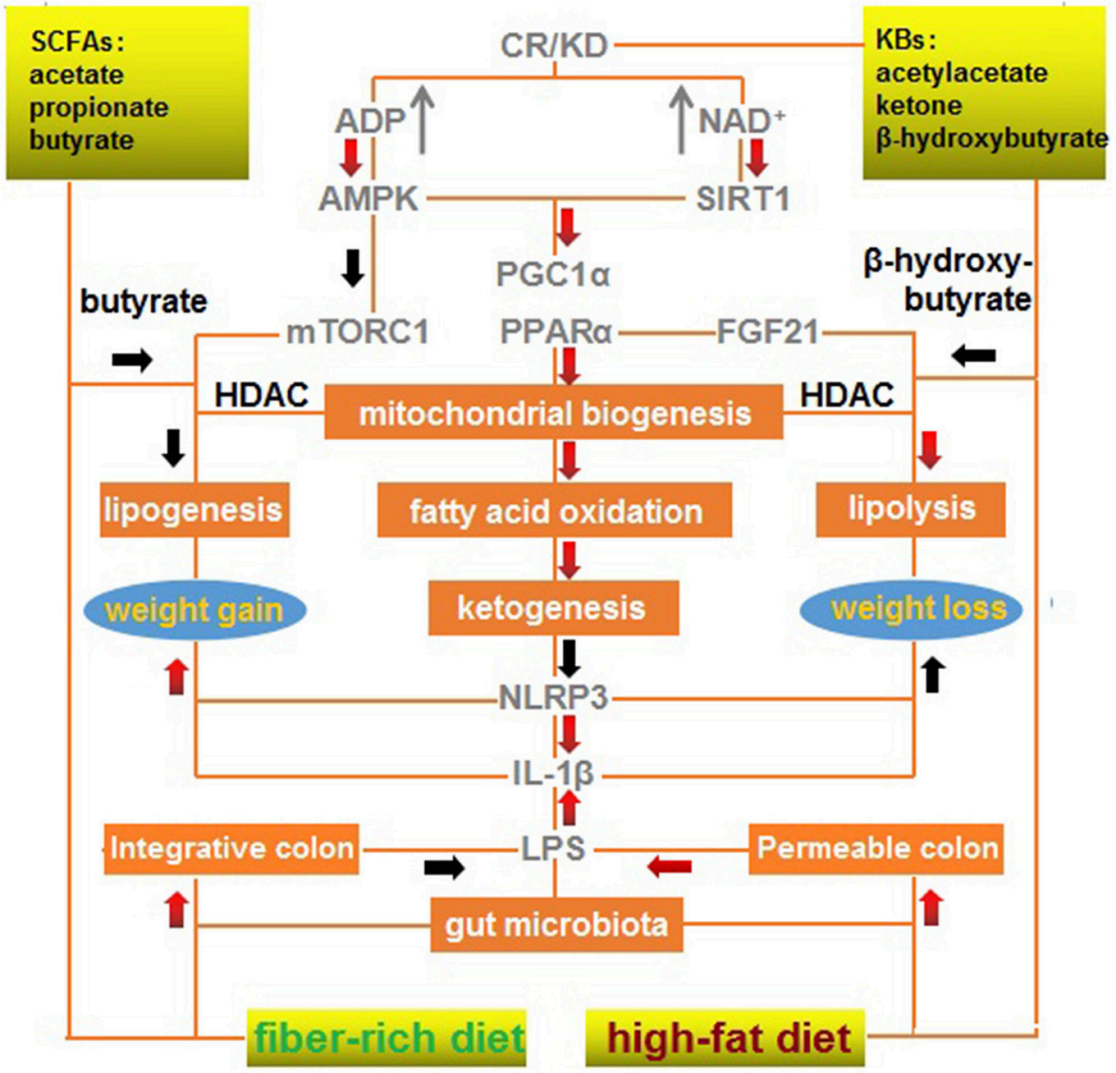
A mechanistic outline of the interactions between a modified fiber-rich ketogenic diet with multiple signaling pathways associated with weight loss. A ketogenic diet mimics calorie restriction to improve mitochondrial function via ketone bodies, resulting in anti-inflammatory effects, and a fiber-rich diet maintains gut homeostasis via short-chain fatty acids, which exert anti-infectious effects. A red arrow represents positive regulation (upregulation/increase); and a black arrow represents negative regulation (downregulation/decrease). CR, calorie restriction; KBs, ketone bodies; KD, ketogenic diet; SCFAs, short-chain fatty acids.

Maintenance of gut microbiota homeostasis is the most critical factor in eliminating inflammatory obesogenic drivers, particularly with regard to gut opportunistic infection and endotoxin-triggered inflammation. SCFAs and KBs, which are structurally and functionally similar, are complementary in restoring gut homeostasis and rectifying mitochondrial dysfunction. An integrative signaling framework responsible for weight loss is summarized in Figure 1. This scheme illustrates a primary effect of CR/KD on mitochondrial biogenesis, fatty acid oxidation, and ketogenesis, as well as concurrent effects of a fiber-rich diet on the integrative colon and low-level LPS and effects of a high-fat diet on the permeable colon and high-level LPS.

Briefly, KD can mimic CR to activate AMPK, SIRT1, PGC-1α, and PPARα to enhance mitochondrial biogenesis, fatty acid oxidation, and ketogenesis. These processes can inhibit NLRP3 and IL-1β that promote weight gain and repress weight loss. Butyrate from a fiber-rich diet can mimic the effects of β-hydroxybutyrate produced by a high-fat diet to inhibit HDAC, promote lipolysis, and repress lipogenesis, resulting in mTORC1 inactivation and FGF21 activation.

## Author Contributions

Q-PZ wrote the manuscript. J-PS, QX, C-QL, and QW critically reviewed the manuscript. All authors read and approved the final version of the manuscript.

### Conflict of Interest Statement

The authors declare that the research was conducted in the absence of any commercial or financial relationships that could be construed as a potential conflict of interest.
